# Early reduction in painful physical symptoms is associated with improvements in long-term depression outcomes in patients treated with duloxetine

**DOI:** 10.1186/1471-244X-11-150

**Published:** 2011-09-20

**Authors:** Edith Schneider, Michael Linden, Harald Weigmann, Thomas Wagner, Deborah Quail, Hans-Peter Hundemer, Ulrich Hegerl

**Affiliations:** 1 Lilly Deutschland GmbH, Medical Department, Bad Homburg, Germany; 2 Research Group Psychosomatic Rehabilitation at the Charité, University Medicine Berlin and the Rehabilitation Centre Seehof, Teltow/Berlin, Germany; 3 Boehringer Ingelheim Pharma GmbH & Co KG, A Medizinische Wissenschaft, Ingelheim am Rhein, Germany; 4 Dept European Medical Information Sciences, Eli Lilly and Co Ltd, Windlesham, UK; 5 Department of Psychiatry, University of Leipzig, Germany

**Keywords:** Depression, painful physical symptoms, non-interventional study, duloxetine

## Abstract

**Background:**

To investigate the association of the change of painful physical symptoms (PPS) after 4 weeks, with the 6-month treatment outcomes of depressive symptoms in patients treated with duloxetine in clinical practice.

**Methods:**

Multicenter, prospective, 6-month, non-interventional study in adult outpatients with a depressive episode and starting treatment with duloxetine. Depression severity was assessed by the clinician (Inventory for Depressive Symptomatology [IDS-C]) and patient (Kurz-Skala Stimmung/Aktivierung [KUSTA]). Somatic symptoms and PPS were assessed using the patient-rated Somatic Symptom Inventory (SSI) and visual analog scales (VAS) for pain items. Association of change in PPS with outcomes of depressive symptoms was analyzed based on mean KUSTA scores (mean of items mood, activity, tension/relaxation, sleep) and achievement of a 50% reduction in the total IDS-C score after 6 months using linear and logistic regression models, respectively.

**Results:**

Of the 4,517 patients enrolled (mean age: 52.2 years, 71.8% female), 3,320 patients (73.5%) completed the study. 80% of the patients had moderate to severe overall pain (VAS > 30 mm) at baseline. A 50% VAS overall pain reduction after 4 weeks was associated with a 13.32 points higher mean KUSTA score after 6 months, and a 50% pain reduction after 2 weeks with a 6.33 points improvement. No unexpected safety signals were detected in this naturalistic study.

**Conclusion:**

Pain reduction after 2 and 4 weeks can be used to estimate outcomes of long-term treatment with duloxetine. PPS associated with depression have a potential role in predicting remission of depressive symptoms in clinical practice.

## Background

Depressive patients frequently report somatic symptoms including painful physical symptoms (PPS) accompanying their depression (mean prevalence 65%) [1]. The causal relationship between pain and depression remains unclear [2-4]. Pain can be a symptom, a cause, or a consequence of depression [2]. Neurobiological evidence suggests that mood and chronic pain are connected via the serotonin and noradrenalin neurotransmitter pathways. A malfunctioning of the descending serotonergic and noradrenergic pathways could allow routine sensory input to be interpreted as uncomfortable or even painful [5,6]. Studies investigating the direction of the association between pain and depression suggest that it is the stress of living with chronic pain that causes depression [7], but there is also evidence that pain develops secondary to depression through increases in pain sensitivity and that high depression scores result in a greater risk of developing chronic pain [8].

In 69% of depressed patients, painful or non-painful physical symptoms were the only presenting complaints in general practice [9]. This can lead to a lack of awareness of depression, missed diagnosis [10,11] and inappropriate treatment [12]. Conversely, failure to treat PPS in depressed patients may adversely impact depression treatment outcomes [13,14]. Recognizing and optimizing the management of pain that commonly coexists with depression may be important in enhancing depression response and remission rates [15]. The presence of severe pain at start of depression treatment has been associated with non-response to antidepressants [16], and a lower overall pain severity score at baseline was associated with higher odds of achieving remission [17].

In a naturalistic clinical trial addressing long-term treatment of PPS and emotional depressive symptoms [18], it was found that the effect of selective serotonin reuptake inhibitors (SSRIs) on PPS was less pronounced than on the emotional symptoms. However, this trial was restricted to SSRIs, and it is argued that for an adequate pain response, substances affecting both serotonin and noradrenaline are necessary [19].

Extensive data support the efficacy of tricyclic antidepressants (TCAs) for the alleviation of pain in chronic pain patients [20], and also the newer serotonin and noradrenaline reuptake inhibitors (SNRIs) duloxetine [14], venlafaxine [21] and milnacipran [22] have shown efficacy in the treatment of pain and depression. Duloxetine is a SNRI with proven efficacy for PPS of depression [14,23,24]. Analyses from short-term trials demonstrated that a greater reduction in pain was associated with a higher probability of remission [14,25]. Furthermore, the efficacy of duloxetine has been also proven for the treatment of painful diabetic neuropathy [26].

The primary research objective of the present 6-month, observational study with duloxetine ('PADRE') was to investigate the association of an early improvement in PPS with long-term changes in depressive symptoms, which could be used as a predictor of long-term treatment outcomes in depression. Such a predictor could be helpful for an early adjustment of treatment to the individual patient [27].

## Methods

### Study Design

The present multicenter, prospective, non-interventional study (F1J-SB-B009) investigated the influence of early changes in PPS in depressed patients on long-term changes in depressive symptoms during treatment with duloxetine in clinical practice over a period of 6 months.

The study was conducted at 693 centers in Germany. Initially, all psychiatrists/neurologists of the Lilly database (about 5000, representing about 70% of office based psychiatrists/neurologists who are involved in pharmacological treatment of depression in Germany) were contacted and finally 693 centers actively participated in the study. Outpatients (age ≥ 18 years) with a depressive episode (according to ICD-10) who were initiated to antidepressive treatment with duloxetine were allowed to enter the study. Treatment patterns were solely at the discretion of the physician and the patient.

The study was approved by the appropriate ethics committee and notified to the German national authority (BfArM, Bundesinstitut für Arzneimittel und Medizinprodukte). Patients provided written consent to the collection and release of anonymized data (according to the Declaration of Helsinki).

Data were collected at baseline (i.e. prescription of duloxetine), after 2 weeks, 1, 3, and 6 months, or at early discontinuation of observation. The study was conducted from August 2005 until December 2007.

### Assessments

Depressive symptoms were assessed using the daily self-rating KUSTA scale (Kurz-Skala Stimmung/Aktivierung - Short Mood/Drive Scale) [28]. A high criteria-related validity is indicated by correlations with other rating scales such as the Hamilton Rating Scale for Depression (HAMD; maximum correlation coefficient: r = 0.93). For the present study, the KUSTA items mood, activity, tension/relaxation and sleep were each rated on a 100 mm Visual Analogue Scale (VAS), and a "mean KUSTA score" determined by calculating the arithmetic mean from the values of these 4 items.

The Inventory for Depressive Symptomatology (IDS) [29] is an instrument for the evaluation of the severity of depression. A validated German translation of the clinician rated version (IDS-C) with 30 items was used [30]. The items address simple, single symptoms rated on a 4-step Likert scale ranging from 0 to 3, with severity levels described in terms relevant to each item. Changes in PPS were assessed by using self-rated VAS for overall pain, headache, shoulder/neck pain, back pain, joint pain, thoracic pain, and abdominal pain.

Changes in painful and non-painful physical symptoms were assessed by the patient-rated Somatic Symptom Inventory (SSI) [31]. The SSI consists of 28 symptoms, each of which is rated on a 5-point Likert scale (1 = "not at all" to 5 = "very much").

The outcome assessments were conducted at the visits in the physician's office, either by the patient (KUSTA, VAS pain, SSI) or by the investigator (IDS-C).

Improvements are indicated by a decrease of the respective scores for IDS-C, VAS Pain and SSI, and by an increase of the Mean KUSTA score.

Demographics and other baseline parameters, treatment decisions, concomitant use of analgesics, hospitalizations for depression, and tolerability data (adverse events [AEs]) were collected.

### Statistics

For this study, about 4,300 patients were planned to be recruited. This sample size allows measurement of changes in pain using the VAS with adequate precision (10% of the standard deviation [SD]) in all subgroups derived from the combination of gender and baseline pain (≤ 30 or > 30 mm VAS [32]). It was assumed that the smallest subgroup, comprising one ninth of the population, would be males with baseline pain > 30 mm, and that 20% of the patients would not provide follow-up data.

Data analyses were performed using SAS version 9.1.3 statistical software. All analyses were exploratory; no confirmatory statistical tests were performed, or statements derived. Continuous variables were summarized using descriptive statistics (number of patients, mean, median, SD, range) and binary or categorical variables using absolute and relative frequencies. A conservative test of whether mean changes over time were statistically significant was done by seeing whether the 95% confidence intervals for the means at the time points overlapped. To address the primary objective, the Mean KUSTA score and whether or not patients had achieved a ≥ 50% reduction in the total IDS-C score at the final visit were analyzed, using linear and logistic regression models, respectively. These models included the following variables:

• Baseline score of the outcome variable

• Gender, age, employment status, whether living alone

• Number of weeks unable to work in the last 12 months

• Currently unable to work

• Duration of depression

• Concomitant psychiatric/somatic diseases

• Baseline psychotropic/permanent pain medication

• Pain symptoms at baseline

• Initial dose of duloxetine

• Overall pain VAS at baseline

• ≥ 50% overall pain VAS reduction at 4 weeks

• ≥ 50% reduction in SSI painful symptoms subscore at 4 weeks

• SSI painful and non-painful symptom subscores at baseline

All of these independent variables were included in a full model and then removed stepwise. Model calculation was repeated with 50% reduction in VAS for overall pain and SSI between baseline and 2 weeks instead of at 4 weeks. Post-hoc, this model was repeated for patients with clinically relevant (> 30 mm) baseline pain only.

The effect of non-painful physical symptoms on outcomes was also investigated using regression methods. Model fit was checked by reviewing plots of residuals for the linear regression and the Hosmer-Lemeshow goodness-of-fit test for logistic models. Sensitivity analyses (last observation carried forward [LOCF], repeated measures) were performed to check the robustness of the primary analysis.

A further post-hoc analysis in patients with > 30 mm pain at baseline examined the predictive value of an early response in depressive symptoms (using the IDS-C score, ≥ 50% reduction after 4 weeks and ≥ 20% reduction after 2 weeks) in conjunction with an early response in pain (≥ 50% reduction in overall pain VAS after 4 weeks and ≥ 20% reduction after 2 weeks) and other possible predictive factors.

Data quality was assured by implementing a data validation plan and double data entry. Data from incomplete scales (i.e. missing values for one or more items) were excluded from statistical analysis for the respective visit and patient.

## Results

### Patients

**Figure 1:**
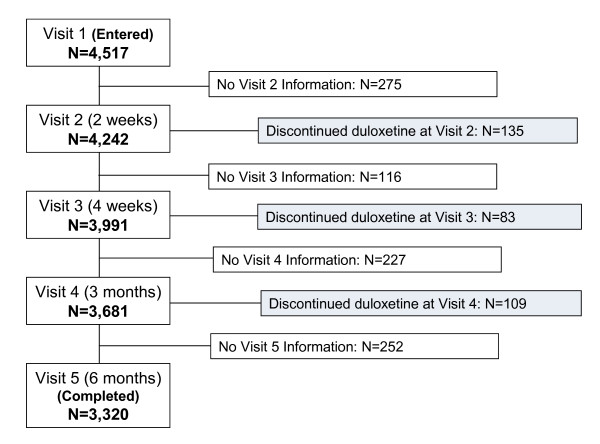
**Patient Flow Chart**.

A total number of 4,517 patients were enrolled into the study, 3,320 patients (73.5%) reached the endpoint at Visit 5 (6-month [see Figure 1]). Reasons for discontinuation could be documented from 514 patients (321 before Visit 5 and 193 at Visit 5). The most frequently reported reasons for discontinuation were patient decision (34.0%, 175 of 514 patients) and AE (23.2%, 119 of 514 patients). Patients' demographics, diagnoses, and medical history are summarized in Table 1.

**Table 1:** Patient Demographics, Diagnosis, and Medical History

Variable	n (%)	Mean (SD)
Age (years; N = 4508)		52.2 (12.7)
Gender: Female (N = 4513)	3241 (71.8)	
BMI (kg/m^2^; N = 4503)		27.1 (5.1)
Living alone (N = 4261)	1113 (26.1)	
Currently unable to work ^a ^(N = 4321)	1708 (39.5)	
Duration of inability to work in the last 12 months (weeks; N = 4140)		6.3 (13.1)
Diagnosis by ICD Code (reported by > 5% of patients; N = 4493)		
Moderate depressive episode (F32.1)	1376 (30.6)	
Recurrent depressive disorder, current episode moderate (F33.1)	1204 (26.8)	
Severe depressive episode without psychotic symptoms (F32.2)	569 (12.7)	
Recurrent depressive disorder, current episode severe without psychotic symptoms (F33.2)	362 (8.1)	
Depressive episode, unspecified (F32.9)	347 (7.7)	
Age at onset of depression (years; N = 4445)		41.5 (14.3)
Time since onset of depression (years; N = 4442)		10.6 (10.7)
Any hospitalization during the last 12 months (N = 4485)	473 (10.5)	
Any suicide attempt during the last 12 months (N = 4473)	102 (2.3)	
Any concomitant psychiatric diseases (N = 4501)	2032 (45.2)	
Most common (> 10% of patients) concomitant psychiatric diseases: ^b^		
Somatoform disorders	1269 (28.2)	
Anxiety disorders/obsessive-compulsive disorders	661 (14.7)	
Further psychiatric diseases	497 (11.0)	
History of antidepressant therapy in the last week (N = 4500) yes	2678 (59.5)	
Most common (> 5% of patients) antidepressant therapies:		
Tricyclic antidepressant	1320 (29.3)	
Selective serotonin reuptake inhibitor	1063 (23.6)	
Noradrenergic and specific serotonergic antidepressant	385 (8.6)	
Selective serotonin and noradrenaline reuptake inhibitor	234 (5.2)	
Patients with overall pain VAS > 30 mm	3525 (80.0)	
Any permanent pain medication (N = 4503)	1453 (32.3)	
Any on-demand pain medication in the last 12 months (N = 4481)	2728 (60.9)	
Any concomitant somatic diseases (N = 4495)	3241 (72.1)	
Most common (> 10% of patients) concomitant somatic diseases: ^b^		
Muscle and skeleton diseases	1514 (33.7)	
Hypertension	1258 (28.0)	
Neurologic diseases	555 (12.3)	
Metabolic diseases	503 (11.2)	
Gastrointestinal diseases	470 (10.5)	
Allergies	467 (10.4)	

BMI = Body mass index; N = Number of patients with available data; n = Number of patients in category; SD = Standard deviation.

^a ^Answer 'yes' to the question 'Is the patient unable to work today?'.

^b ^As selected from the check list.

### Medication

At study entry, 45.8% of the patients started duloxetine treatment as their initial medication for depression, 50.3% were switched due to inadequate effectiveness of their previous medication. The remainder named different reasons for their switching to duloxetine. The initial duloxetine dose was 30 mg/d in 72.9% of the patients. At 2 and 4 weeks, 64.8% and 73.0% of the patients received 60 mg/d respectively.

### Efficacy Results

#### Depressive Symptoms

The mean KUSTA score continuously increased over time (p < 0.05). Correspondingly, the IDS-C total score continuously decreased over time (p < 0.05). The development over time of all individual KUSTA items was similar to that of the mean KUSTA score. Details on the mean KUSTA score and IDS C score are given in Table 2.

**Table 2:** Descriptive Statistics of Efficacy Variables (KUSTA, IDS-C, Pain VAS, SSI) Over Time

Variable	Baseline (N = 4517)	2 Weeks (N = 4242)	4 Weeks (N = 3991)	3 Months (N = 3681)	6 Months (N = 3320)
**Mean KUSTA score **^a^**, mean (SD)**	25.2 (16.8)	34.4 (20.3)	43.3 (23.3)	53.1 (24.7)	58.9 (25.9)
**IDS-C total score, mean (SD)**	39.2 (12.4)	31.8 (13.0)	25.2 (12.8)	19.9 (12.6)	16.1 (11.9)
**VAS (mm), mean (SD)**					
Overall pain	55.0 (26.6)	44.9 (25.5)	39.1 (25.6)	34.2 (25.4)	30.5 (25.4)
Headache	40.8 (31.7)	34.3 (29.7)	30.1 (28.2)	27.2 (27.2)	23.7 (25.6)
Back pain	50.3 (31.7)	40.8 (30.1)	35.9 (28.9)	32.9 (28.5)	29.7 (27.9)
Joint pain	48.5 (32.3)	38.8 (30.0)	34.3 (28.7)	31.4 (28.4)	28.4 (28.3)
Shoulder/neck pain	43.9 (32.8)	34.5 (29.6)	30.2 (28.4)	27.7 (28.1)	24.5 (27.0)
Chest pain	24.8 (28.7)	19.7 (25.1)	17.0 (23.1)	15.8 (22.5)	14.5 (21.5)
Abdomen pain	24.4 (28.4)	20.2 (24.8)	17.3 (23.0)	16.6 (22.8)	14.5 (20.9)
**≥ 30% reduction in VAS overall pain:** All patients, n (%)	NA	1279 (31.9)	1771 (46.9)	1968 (56.2)	1949 (61.9)
Females, n (%)	NA	913 (31.8)	1277 (47.2)	1419 (56.4)	1392 (61.6)
Males, n (%)	NA	366 (32.1)	494 (46.0)	549 (55.6)	557 (62.6)
Patients with > 30 mm in VAS overall pain at baseline	NA	1060 (32.8)	1492 (48.8)	1689 (59.4)	1664 (65.4)
**≥ 50% reduction in VAS overall pain:** All patients, n (%)	NA	705 (17.6)	1129 (29.9)	1427 (40.7)	1516 (48.1)
Females, n (%)	NA	500 (17.4)	811 (30.0)	1019 (40.5)	1075 (47.6)
Males, n (%)	NA	205 (18.0)	318 (29.6)	408 (41.3)	441 (49.6)
Patients with > 30 mm in VAS overall pain at baseline	NA	539 (16.7)	910 (29.8)	1193 (42.0)	1273 (50.1)
**SSI, mean (SD)**					
Total score	2.47 (0.69)	2.20 (0.68)	2.00 (0.66)	1.84 (0.66)	1.72 (0.65)
Painful symptoms	2.76 (0.88)	2.43 (0.84)	2.23 (0.81)	2.04 (0.79)	1.90 (0.77)
Non-painful symptoms	2.38 (0.69)	2.13 (0.67)	1.92 (0.65)	1.77 (0.66)	1.65 (0.64)

KUSTA = Kurz-Skala Stimmung/Aktivierung; N = Total number of available patients (number of patients with available data varied depending on variable and visit); SD = Standard deviation. NA = Not applicable; SD = Standard deviation; SSI = Somatic Symptom Inventory; VAS = Visual analogue scales.

^a ^Mean of KUSTA scores items mood, activity, tension/relaxation and sleep,, range: score 0-100

#### Painful and Non-painful Physical Symptoms

VAS pain scores and SSI scores improved continuously over time during the study (p < 0.05) as shown in Table 2.

Categorical analyses of the proportions of patients with a reduction of ≥ 30% or ≥ 50% in VAS overall pain from baseline to 6 months are shown in Table 2. There was a consistent decrease in the number of patients with a VAS overall pain score of > 30 mm during the course of the study, from 80.0% at baseline to 43.9% at Month 6.

#### Primary Analysis

The linear regression analysis showed that in the applied model, a 50% reduction in overall pain VAS during the first 4 weeks had the strongest association (F-value = 158.6; p < 0.0001) of all variables assessed with the mean KUSTA score at 6 months. For patients with a ≥ 50% reduction in overall pain VAS during the first 4 weeks, the mean KUSTA score at 6 months was estimated to be 13.32 points higher than for patients without a ≥ 50% reduction. These results were supported by sensitivity analyses based on LOCF and repeated measures approaches. The results were similar for patients with baseline pain > 30 mm. All variables with a statistically significant effect in the regression analysis of the mean KUSTA score are given in Table 3.

**Table 3:** Statistically Significant Variables at Baseline and after 4 Weeks in the Regression Analysis of the Mean KUSTA Score at 6 Months

a) All patients (N = 2574)			
**Variable**	**F-value**	**p-value**	**Estimate ^a^**

≥ 50% reduction in overall pain VAS during the first 4 weeks	158.6	< 0.0001	13.32
Number of weeks unable to work in the last 12 months	40.9	< 0.0001	-0.23
Overall pain VAS at baseline (per 20 mm)	33.9	< 0.0001	-2.34
Any concomitant somatic disease at baseline	30.5	< 0.0001	-5.86
SSI non-painful symptoms subscore at baseline	18.0	< 0.0001	-3.49
Living alone	13.8	< 0.001	-3.89
≥ 50% reduction in the SSI painful symptoms subscore during the first 4 weeks	11.0	< 0.001	6.22
Any baseline psychotropic medication	7.9	0.005	-2.93
Duration of depression (years)	6.1	0.014	-0.12
Mean KUSTA at baseline (per 20 mm)	5.6	0.018	1.47

**b) Patients with baseline pain VAS > 30 mm (N = 2053)**			

**Variable**	**F-value**	**p-value**	**Estimate ^a^**

≥ 50% reduction in overall pain VAS during the first 4 weeks	151.4	< 0.0001	14.74
Number of weeks unable to work in the last 12 months	40.2	< 0.0001	-0.25
Any concomitant somatic disease at baseline	22.1	< 0.0001	-5.98
SSI non-painful symptoms subscore at baseline	16.0	< 0.0001	-3.54
Overall pain VAS at baseline (per 20 mm)	15.0	< 0.001	-2.50
Living alone	13.3	< 0.001	-4.24
Mean KUSTA at baseline (per 20 mm)	9.9	0.002	2.36
≥ 50% reduction in the SSI painful symptoms subscore during the first 4 weeks	8.7	0.003	5.84
Age	4.1	0.044	-0.09
Duration of Depression (years)	3.9	0.048	-0.10

^a ^For binary outcomes (yes/no) the given estimate reflects the average impact on the KUSTA score if the respective factor is present ("yes") compared to the situation where it is not present ("no"), e.g. in this model the mean KUSTA score for a patient living alone is estimated to be 3.89 points lower than for patient not living alone. For continuous outcomes, the estimate reflects the difference in the KUSTA score for each unit increase of the respective covariate, e.g. in this model for each additional year of duration of depression the mean KUSTA score is reduced by 0.12 points.

In the logistic regression analysis of the response rate in the IDS-C total score (50% reduction [yes/no]), the IDS-C total score at baseline and the change in the overall pain VAS during the first 4 weeks had the strongest effect (p < 0.0001) among the factors included in the model.

The odds ratio for the change in the overall pain VAS during the first 4 weeks was 3.00 (95% CI: 2.41-3.75). This indicates that for patients with a ≥ 50% reduction in their overall pain VAS during the first 4 weeks, the odds of achieving a 50% reduction in the IDS-C total score after 6 months is 3 times higher than for those who did not. Expressed as relative risks, the probability of achieving a 50% reduction in the IDS-C total score was 1.45 times higher for those patients with an early ≥ 50% reduction in their overall pain VAS.

Further statistically significant factors were similar to those seen in the analysis of the mean KUSTA score.

When performing the model based on 2-week data, the ≥ 50% reduction in overall pain VAS during the first 2 weeks was identified as a relevant factor in influencing the outcome of depressive symptoms after 6 months. Two-week data were associated with a 6.33 point improvement in the 6-month mean KUSTA score, compared with patients without a ≥ 50% pain reduction.

**Figure 2:**
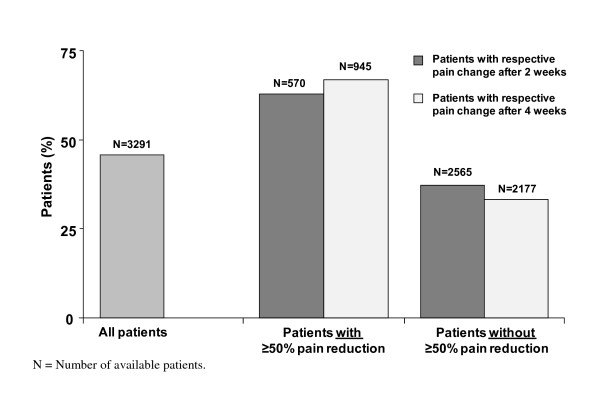
**Remission Rates (IDS-C ≤ 12) after 6 Months of Treatment with Duloxetine**.

The remission rate based on IDS-C (total score ≤ 12) after 6 months was 45.9% for all patients. In a regression analysis, pain reduction (decrease in VAS overall pain of ≥ 50%) during the first 4 weeks was the factor most strongly associated with remission rate (p < 0.0001), with an odds ratio of 2.90 (95% CI: 2.38-3.52). The relationship between an early pain reduction after 2 and 4 weeks and the remission rate after 6 months is shown in Figure 2. The remission rate in patients with an early pain reduction after 2 weeks (62.8%) was almost as high as in patients with a pain reduction after 4 weeks (66.9%).

In a post-hoc analysis, the early reduction of depressive symptoms measured with the IDS-C scale was added to the linear regression models. Using the reductions during the first 4 weeks in patients with clinically relevant pain (> 30 mm VAS) at baseline, the ≥ 50% reduction in overall pain VAS (F = 94.5, p < 0.0001, estimate = 11.39) and the ≥ 50% reduction in IDS-C total score after 4 weeks (F = 91.8 p < 0.0001, estimate = 11.28) showed the strongest associations with 6-month KUSTA depressive outcomes. An analysis based on the 2-week results with a 20% reduction criterion also showed a strong association of the reduction in overall pain VAS (F = 35.9, p < 0.0001, estimate = 6.52) and the IDS-C total score (F = 29.4, p < 0.0001, estimate = 5.89) with 6-month KUSTA depression outcomes.

### Safety Results

During the observation period, 132 patients (3.1%) were hospitalized due to depression, the median duration was 23 days (range: 1 to 153 days).

At least one treatment emergent adverse event (TEAE) was reported by 741 patients (17.2%). The most frequently affected system organ classes (at least 3% of patients with an event) were gastrointestinal disorders (395 patients [9.2%]), psychiatric disorders (200 [4.6%]), nervous system disorders (153 [3.5%]), and skin and subcutaneous tissue disorders (131 [3.0%]). The only TEAE that was reported by more than 3% of patients was nausea (226 patients [5.2%]).

Serious TEAEs were reported by a total of 34 patients (0.79%). The only serious TEAEs reported by more than 2 patients were depression (6 patients [0.14%]) and diarrhea (3 [0.07%]). Serious TEAEs included one report of suicidal ideation; however, no suicide or suicide attempt was reported. Two patients had fatal TEAEs: renal cancer; cerebral hemorrhage.

At each post-baseline visit, mean weight had decreased compared to baseline, but mean decreases were not greater than 0.23 kg below baseline at any time point.

## Discussion

In the PADRE study, 80% of the depressed patients had moderate to severe overall pain at baseline. For all patients, the mean overall pain VAS score was 55.0 mm. This high pain intensity could partly result from the fact that depressed patients with pain syndromes could be overrepresented in this study because of the proven analgesic efficacy of duloxetine [14,23]. However, the observed prevalence of pain in depressed patients in the PADRE study is in line with the literature reporting prevalence rates of 56% [33], 65% [1] and 88% [34].

For 72.1% of the PADRE population, concomitant somatic diseases were documented most frequently 'muscle and skeleton diseases' (33.7%). The high prevalence of patients suffering from pain conditions may raise concern that common depression rating scales overestimate depressive symptoms in these patients, as they may score higher on somatic items because of their pain rather than because of their mood. However, Poole et al. [35] showed a good correlation of a commonly used depression rating scale that includes somatic items (Beck Depression Inventory-II) with a structured clinical interview for DSM-IV Axis Disorders (SCID) which represents a gold standard for assessment of depressive symptomatology.

The main finding of our study is that early change in pain severity was strongly associated with a long-term reduction of depressive symptoms according to the mean KUSTA score. Pain responders also had a higher chance of achieving a 50% reduction in the IDS-C total score after 6 months.

This association of an early improvement in pain with long term depression outcomes was already seen after 2 weeks, albeit, to a smaller extent than after 4 weeks. This is remarkable, considering that 72.9% of the patients started duloxetine treatment with a dose of 30 mg/day, which is lower than the recommended starting and maintenance dose.

Patients with a VAS pain reduction of ≥ 50% after 2 and 4 weeks, showed also higher remission rates after 6 months than patients without a ≥ 50% pain reduction (Figure 2).

For clinicians, it is of interest whether or not early improvement of pain is a better predictor of the long-term outcome to antidepressant treatment than early improvement of depressive symptoms. Therefore, post-hoc analyses were performed including early response in depressive symptoms after 2 and 4 weeks (as measured with IDS-C total score). Results showed that an early pain response had similar predictive value compared to early depression response for long term depressive outcomes as measured with the KUSTA scale.

The main results of the PADRE study are in contrast to a recent publication [36], reporting a very low predictive association between analgesic and antidepressant responses in six placebo-controlled trials assessing the efficacy of duloxetine in patients with major depressive disorder. However, the studies used in these post-hoc meta-analyses were not designed to assess the relationship between antidepressant and analgesic response, and there were only few data points available for early response assessment.

Other studies support the association between early improvement in pain and long-term antidepressant response [15,37]. Pooled data from two 9-week randomized, double-blind duloxetine studies showed that the remission rate for pain responders (improvement in VAS overall pain from baseline to last observation ≥ 50%) was twice that observed for pain non-responders (36.2% vs. 17.8%, p < 0.001). Improvements in pain severity were also related to improved quality of life and improved clinician- and patient-rated global health outcomes [14]. A secondary analysis of a 12-week open-label trial with duloxetine in 249 patients [25] found similar results. Patients who experienced clinically important pain reduction in the first week of duloxetine treatment were significantly more likely to reach remission at endpoint than the patients without this pain reduction (64.0% vs. 35.6%, p < 0.001).

The results of the PADRE study are of interest in the context of other recent research [27,38-42]. Evidence was provided questioning the belief that antidepressant response usually appears with a delay of several weeks, and new evidence continues to accumulate that individual improvement within the first 2 weeks is a key predictor of treatment response. Lack of early response in depression symptom subscales was highly predictive of a lack of sustained remission [41]. A lack of improvement during the first 2 weeks of therapy may indicate that changes in depression management should be considered earlier than conventionally thought [32]. Results from PADRE also suggest that early improvements in concomitant PPS measured with simple VAS scales should be considered in treatment decisions during the initial 2-4 weeks of a new antidepressive treatment. The results of PADRE could also be important for patients with generalized anxiety disorder with or without comorbid MDD, as the clinical relevance of PPS and resulting functional impairment has been reported [43,44].

As this was a large observational study in daily clinical practice, several methodological limitations such as the absence of monitoring or a high number of patients lost to follow-up were unavoidable and may lead to concerns with regard to data quality. However, this non-interventional approach reflects current treatment of patients with MDD by office-based psychiatrists and should therefore allow generalization of our results to clinical practice. Perhaps the most serious shortcoming of non-interventional trials is selection bias because of absence of randomization. Another limitation of the study is the lack of a control group, as only duloxetine-treated patients were included.

## Conclusions

Pain is a frequent and often severe concomitant symptom in depressive patients in clinical practice. Pain reduction after 2 and 4 weeks can be used to estimate long-term outcomes regarding successful antidepressive treatment with duloxetine.

The present results emphasize the importance of PPS associated with depression because of their potential role in predicting and achieving depressive symptom remission.

## Declaration of Competing interests

Edith Schneider, Harald Weigmann, Thomas Wagner, Deborah Quail, and Hans-Peter Hundemer are employees of Eli Lilly or Boehringer Ingelheim.

Ulrich Hegerl is speaker/advisory board member for Lilly, Lundbeck, GlaxoSmithKline and Bristol-Myers Squibb.

Michael Linden is consultant and speaker/advisory board member for Lilly and Lundbeck.

This research was funded by Lilly Deutschland GmbH and Boehringer Ingelheim Pharma GmbH & Co KG.

## Authors' contributions

ES, UH, ML, TW, and HPH have participated in the study design, interpretation of results, and writing of the manuscript. DQ carried out the statistical analysis, participated in the interpretation of results, and writing of the manuscript.

All authors read and approved the final manuscript.

## Pre-publication history

The pre-publication history for this paper can be accessed here:

http://www.biomedcentral.com/1471-244X/11/150/prepub
